# Patient-controlled admissions to inpatient care: A twelve-month naturalistic study of patients with schizophrenia spectrum diagnoses and the effects on admissions to and days in inpatient care

**DOI:** 10.1186/s12913-021-06617-8

**Published:** 2021-06-24

**Authors:** Maria Skott, Natalie Durbeej, Maria Smitmanis-Lyle, Clara Hellner, Emelie Allenius, Sigrid Salomonsson, Tobias Lundgren, Nitya Jayaram-Lindström, Alexander Rozental

**Affiliations:** 1 Centre for Psychiatry Research, Department of Clinical Neuroscience, Karolinska Institutet, 117 63 Stockholm, Sweden; 2 Stockholm County Council, Stockholm Health Care Services, Stockholm, Sweden; 3 Child Health and Parenting, Department of Public Health and Caring Sciences, Uppsala University, Uppsala, Sweden; 4 Great Ormond Street Hospital Institute of Child Health, University College London, London, UK

**Keywords:** Schizophrenia spectrum diagnoses, Inpatients, Patient-controlled admissions, Patient participation, Inpatient care

## Abstract

**Background:**

Patients with schizophrenia spectrum diagnoses have a poor prognostic outlook and rates of recovery. Inpatient care is common, but the decision to initiate such care is not solely up to the patient but also influenced by the healthcare providers. Recent ideas about shared decision-making however challenges this idea. Patient-Controlled Admissions (PCA) refers to a care model where a patient signs a contract that allows the decision for admission into inpatient care to be transferred onto the patient.

**Methods:**

In Region Stockholm’s public healthcare PCA was introduced to patients with schizophrenia spectrum diagnoses deemed to have the greatest care needs. Outcomes of a 12-month naturalistic within-group follow-up was analyzed using Wilcoxon signed-rank test.

**Results:**

In total, 56 patients fulfilled the study’s inclusion criteria, with between 20 to 42 patients having complete data and being able to analyze statistically, depending on the variable. Number of admissions, inpatient days, number of involuntary admissions, and involuntary admission days decreased, but only significantly so for inpatient days, *p* < .01 (a mean reduction of 11.5 days). Neither self-rated well-being, as assessed using the EQ5D-3L, or a clinician-administered rating of overall health status, the Clinical Global Impression Scale, demonstrated a significant change.

**Conclusions:**

The use of PCA points towards a trend in decreased hospitalization for patients with schizophrenia spectrum diagnoses, although this needs to be explored further in larger samples and over a longer follow-up.

## Introduction

Schizophrenia spectrum diagnoses have a lifetime prevalence of 0.5–1.0% in the general population and constitutes a group of highly pervasive psychiatric disorders caused by genetic and environmental factors [1]. Characterized by both positive symptoms such as delusions and hallucinations and negative symptoms like anhedonia, the prognostic outlook is usually quite poor [2]. Full recovery occurs in less than 14% of all patients within five years of the first psychotic episode and only 16% experience a late-onset recuperation [3, 4], reflecting an often chronic and disabling trajectory. Schizophrenia spectrum diagnoses typically require long-term monitoring by healthcare providers and regular visits to outpatient clinics in order to oversee both medication and overall functioning [5]. Sometimes patients also have novel psychotic episodes or a deterioration in well-being that warrant hospitalization [6]. Since the end of institutionalized care of severe psychiatric disorders, admissions to inpatient care are heavily restricted and usually determined on a case-by-case basis [7]. However, recent ideas of shared decision-making challenge this care model in favor of patient participation [8]. Instead of relying on the judgment of healthcare providers when seeking inpatient care, examples of allowing patients to admit themselves when necessary have been implemented in several countries, e.g., the Netherlands, Norway, Denmark, and Sweden [9–13]. The basic concept of such Patient-Controlled Admissions (PCA) is to transfer responsibility and decision-making regarding hospitalization to the patient, facilitating empowerment and responsibility for the ongoing condition [14]. By being able to admit oneself when needed, patients with schizophrenia spectrum diagnoses are believed to become more successful in recognizing early warning signals and averting potential crises, thereby preventing relapse and the prolongation of a psychotic episode and mental distress [15]. Increasing autonomy in this way is also hypothesized to improve such aspects as quality of life [16].

One early study that investigated the effects of PCA comes from Norway [9], where a total of 53 patients (75.5% schizophrenia/other psychotic disorders) were randomized to PCA or Treatment as Usual (TAU). Here, as in most cases of PCA, patients given the opportunity to admit themselves needed to sign a contract allowing up to five inpatient days, sometimes followed by a quarantine period of two weeks. The results at the four-month follow-up demonstrated that, for patients with PCA, 10.8% of their usual inpatient days became self-referred. However, in terms of hospitalization, that is, admissions and days in inpatient care, no statistically significant difference was detected between the two conditions. At the 12-month follow-up [17], 77% of the patients in PCA were using the opportunity for self-referrals, although both conditions exhibited a decrease in inpatient days, with no statistically significant difference between them. Meanwhile, in a Danish prospective study of 2532 patients, PCA (*n* = 422; 62.8% with schizophrenia spectrum disorder) was matched to TAU (*n* = 2110), based on medical records [10]. At the 12-month follow-up there was a significant difference between PCA and TAU for number of admissions. A closer inspection of each condition revealed that where was an increase in number of admissions for patients with PCA, compared to a decrease for patients receiving TAU. In both cases involuntary admissions and coercive measures decreased, but only PCA increased their medication use. Similarly, a Norwegian study of 57 patients (28.1% schizophrenia spectrum diagnoses), recently presented similar findings [18]. In this four-year pre-post naturalistic design, the total number of admissions increased by 145%, while inpatient days decreased by 44.3%. However, the authors emphasize that it is difficult to rule out regression to the mean effects given the lack of a comparison condition.

In sum, previous research on PCA for patients with schizophrenia spectrum diagnoses is a bit mixed. Evidence suggests that PCA could have some effects in terms of an increased number of admissions to inpatient care and possibly a decrease in inpatient days. Other potential advantages might be fewer coercive measures and an increased number of reimbursed medications. Given the lack of statistically significant differences when compared to TAU, it cannot be confirmed that it is PCA per se that generates these effects. Nonetheless, PCA does not seem to affect patients negatively, at least not in terms of hospitalization. Instead, findings indicate that its use resembles a more traditional care model, but with the possible advantage of reducing coercion and perhaps improving the patients’ motivation to seek help. This is corroborated by other examples of implementing PCA for patients with anorexia nervosa [13], and personality disorders who self-harm and are suicidal [11, 12]. These studies were able to show comparable results for number of admissions, inpatient days, and coercive measures, demonstrating no statistically significant differences to current practice when it comes to hospitalization, but a reduction in actions involving coercion, e.g., forced medication, seclusion, and restraint. Further research is however needed in order to better understand the benefits and limitations of PCA. In particular, evaluating its outcomes in additional settings within healthcare is important to establish its utility at different locations.

The use of PCA was implemented in Sweden in Region Stockholm’s public healthcare in 2014, inspired by the initiative in Norway [9, 17]. In total, six inpatient wards have been involved and offer PCA to patients with schizophrenia spectrum diagnoses which are deemed to have the greatest care needs. The current study aims to examine how the use of PCA has affected number of admissions to inpatient and involuntary care as well as inpatient and involuntary admission days. It also aims to investigate its impact on self-report measures of well-being and a clinician-administered rating of overall health status. The current study intends to replicate and extend the present knowledge of PCA by using naturalistic data from Region Stockholm’s public healthcare, investigating the following research questions:

- For patients with schizophrenia spectrum diagnoses offered PCA, what are their characteristics in terms of demographics and primary diagnosis?
- What are the effects on hospitalization (i.e., number of admissions to inpatient and involuntary care as well as inpatient and involuntary admission days)?
- What are the effects on self-report measures of well-being and a clinician-administered rating of overall health status?

## Methods

### Study design

A naturalistic design was utilized to investigate the effects of PCA for patients having been assigned a contract and consenting to be part of the study. This involved all of the six inpatient wards within the Region Stockholm’s public healthcare that offered PCA to patients with schizophrenia spectrum diagnoses at the time, and who had collected data on their patients during a 12-month period prior to and following the administration of the contracts. In other words, any patient with a contract and giving their consent to participate in the study, with data available from one year before the contract (pre) and after its implementation (post) were analyzed. This allowed for a within-group follow-up on the effects of PCA, but no between-group analysis given the lack of a control condition. The following variables were used as the main outcomes: a) number of admissions to inpatient care, b) number of involuntary admissions, c) number of days in inpatient care, and d) number of days in involuntary care. The data collection period spanned from the introduction of PCA in 2014 (i.e., the first possible data point), to the final contract signed with a patient, which occurred on October 21, 2018. This means that the last available data point was on October 21, 2019 (i.e., considering a twelve-month follow-up period). This latter date marked the implementation of a new version of PCA in Region Stockholm, which differs somewhat from the one described here and includes all patient groups (see the discussion for an overview). Data was collected via Region Stockholm’s public healthcare’s digital patient records system, TakeCare, where healthcare providers manually enter data for number of admissions to inpatient and involuntary care, inpatient and involuntary admission days, and self-report measures of well-being, and a clinician-administered rating of overall health status.

### Setting

Six inpatient wards provided data in the current study, together with an equal number of outpatient clinics, which works as section pairs (one outpatient clinic is connected to an inpatient ward), all being located within Region Stockholm’s public healthcare and being comprised of the municipalities Stockholm and Ekerö (*k* = 3), Solna [1], Danderyd [1], and Sollentuna [1]. This consists of 93 inpatient beds (ranging from 13 to 28 beds), six of which are specifically designated to PCA, i.e., one at each inpatient ward. The healthcare providers include medical doctors (with/without specialist training in psychiatry), nurses (with/without specialist training in psychiatry), physiotherapists, mental health workers, counsellors, psychologists, occupational therapists, and students (e.g., assistant physician undergoing basic or advanced level training).

### Participants and procedure

Patients were deemed eligible to receive a contract and start using PCA if they fulfilled the following inclusion criteria: 1) have a schizophrenia spectrum diagnosis, 2) have had at least one period of inpatient care, 3) have either a history of recurrent care needs or be a patient with a first episode of psychosis, 4) have an up-to-date care plan that includes strategies on how to manage any potential substance abuse, 5) understanding and being motivated to use the contract and be involved in one’s own care, and 6) have an ongoing contact with healthcare providers at the inpatient and outpatient wards. In each case the guiding principle was to assign contracts to those patients regarded to have the greatest care needs, decided in joint discussions between healthcare providers at the inpatient and outpatient wards.

Patients eligible to receive a contract and start using PCA were informed about the conditions for its use, both verbally and in text. The fundamentals of the contract were based on the outline and experience from similar procedures in Norway [9, 17]. This involved being able to contact and ask to be admitted to the inpatient ward that was connected to their outpatient clinic (i.e., section pair). In case the inpatient bed specifically designated for PCA was available, the patient could come in and use it. In case it was unavailable, there was a queue system in place. However, if a more acute admission was needed, i.e., regular intake, usual procedures at the inpatient ward took place. When PCA is being utilized, a registered nurse completed a risk assessment before admitting the patient. This involved assessing possible suicidal ideation and violent behavior. If there was an increased risk, regular intake to inpatient care was made. The maximum length of PCA was five days, but should a longer inpatient period be required, an assessment of her condition was made and the patient was transferred to regular intake. During hospitalization, focus is on the needs of the patient and consultation with a psychiatrist was possible, but no formal instruction on what to offer patients in inpatient care existed, i.e., the same procedures at the inpatient wards. The patient was later discharged by a registered nurse. However, if the condition of the patient worsened, involuntary care could be employed. Compared to other cases of PCA (c.f., [17]), a quarantine period has not been used within Region Stockholm’s public healthcare, i.e., patients did not have to wait before being able to admit oneself again and the number of admissions per month were not restricted.

In case a patient wanted to start using PCA, the contract was signed by the patient and the healthcare providers at the inpatient and outpatient clinics and attached to the patient records. A copy of the contract was also handed out to the patient to bring home, which included all of the details about its use. On the same occasion, the patient was also informed about the possibility to be included in the current study and to sign a separate consent form if interested. In other words, a patient could receive a contract but decline to participate in the research. The consent form was also copied, included in the patient records, and given to the patient for safekeeping. After 12 months, a follow-up was then performed together with the patient, including self-report measures and a clinician-administered rating. On this occasion the contract was also renewed for another year in case the patient wanted to. An overview regarding the characteristics of the 56 patients that were in the end included in the current study can be found in Table 1, while descriptive statistics with regard to symptom severity can be found in Table 5.

**Table 1:** *Patient Characteristics (n = 56)*

Gender: *n* (*%* Men)	30 (53.6)
Age: *M* (*SD*)	46.2 (14.3)
Deceased	1 (1.8%)
Civil status	
Unmarried	43 (76.8%)
Married	6 (10.7%)
Divorced	6 (10.7%)
Children: *n* (% Yes)	5 (8.9%)
Schizophrenia spectrum diagnoses according to ICD-10: *n* (*%*)^a^	
F20.0: Paranoid schizophrenia	13 (23.2%)
F20.1: Hebephrenic schizophrenia	1 (1.8%)
F20.3: Undifferentiated schizophrenia	1 (1.8%)
F20.9: Schizophrenia, unspecified	3 (5.4%)
F22.0: Delusional disorder	3 (5.4%)
F23.8: Other acute and transient psychotic disorder	1 (1.8%)
F25.0: Schizoaffective disorder, manic type	1 (1.8%)
F25.2: Schizoaffective disorder, mixed type	1 (1.8%)
F25.9: Schizoaffective disorder, unspecified	13 (23.2%)
F29.9: Unspecified nonorganic psychosis	19 (33.9%)

*ICD-10* International Statistical Classification of Diseases and Related Health Problems – Tenth Revision

^a^Refers only to the patients’ primary diagnoses, i.e., schizophrenia spectrum diagnoses

The procedures surrounding PCA within Region Stockholm’s public healthcare were coordinated by the Centre for Psychiatry Research at the Department of Clinical Neuroscience at Karolinska Institutet, which is a joint research centre run by Karolinska Institutet and Region Stockholm’s public healthcare. This coordination comprised supporting all healthcare providers with regular training and supervision, that is, a half-day workshop for the assigned coordinators from each clinic, and a half-day workshop for the rest of the healthcare providers, as well as making recurrent visits for additional encouragement and help. The coordinators at the clinics were reimbursed for 10% of their working hours in order to implement PCA, and once a month the coordinators also met with a patient representative who is employed by the Region Stockholm’s public healthcare. However, no systematic attempt at ensuring adherence or fidelity was conducted given the naturalistic study design.

### Measures

Using Region Stockholm’s public healthcare’s digital patient records system variables such as number of admissions to inpatient and involuntary care as well as inpatient and involuntary admission days were recorded continuously for each patient with a contract and later exported for analysis. All inpatient wards were instructed on how to enter this information correctly, including the use of coding schedules specifically developed for PCA that made it possible to track only those with a contract. In addition to data on hospitalization, the patients also completed self-report measures of well-being in relation to signing the contract as well as at the 12-month follow-up, followed by a clinician-administered rating of overall health status. The former consisted of the Swedish version of the EQ5D-3L, which is a five-item questionnaire that asks respondents to classify their current health status on five separate dimensions; mobility, self-care, usual activities (e.g., family), pain/discomfort, and anxiety/depression [19]. Each item is subsequently checked in relation to its severity level, e.g., “I am not anxious or depressed”, “I am moderately anxious or depressed”, and “I am extremely anxious or depressed” (Item 5). The EQ5D-3L is a widely used self-report measure of health-related well-being, which can be utilized to compare patients to population norms and to estimate gains in so-called quality-adjusted life years after treatment. It also asks the patient to rate one’s current health status using a Visual Analogue Scale (VAS) that ranges from zero (“Worst imaginable health state”) to 100 (“Best imaginable health state). The latter consisted of a clinician-administered rating of overall health status, the Clinical Global Impression Scale (CGI [20];). This one-item rating scale ranges from one to seven and asks the healthcare providers to determine the severity of psychopathology for a patient at the onset of treatment or during a clinical interview (referred to as the CGI-S); “Normal, not at all ill” [1] to “Among the most extremely ill patients” [7]. The CGI can also be used to assess recovery during treatment. In this case, “Very much improved since the initiation of treatment” [1] to “Very much worse since the initiation of treatment” [7] (referred to as the CGI-I).

After data was exported for all patients with a contract, consent forms were checked to ensure that only those explicitly consenting to be part of research were included in the analyses and presented in the current study.

### Statistical analysis

Data was examined for violations of normality using the Shapiro-Wilk test and inspections of kurtosis and skewness. Significant results suggest that data is not normally distributed and that non-parametric rather than parametric tests are preferred for the subsequent analyses. Similarly, if estimates of kurtosis and skewness are high or low, this indicate that the distribution of data might be violating assumptions regarding normality. In the current study, the Shapiro-Wilk test was significant for all variables. Number of admissions to inpatient and involuntary care as well as inpatient and involuntary admission days were all positively skewed and demonstred a leptokurtic distribution, meaning that most of the values were close to zero, with a few outliers, i.e., a small number of patients who had very high care needs. With regard to the EQ5D-3L, EQ5D VAS, CGI-S, and CGI-I, data was however negatively skewed and platykurtic, meaning that most values were at the top end, i.e., higher values, including relatively few outliers at the bottom end.

Given the non-parametric nature of the data, Wilcoxon signed-rank test were used to investigate the change on each variable from one year before the contract (pre) to one year after its initiation (post). This is the equivalence of paired samples *t*-test, but for data that is not assumed to be normally distributed. However, because the CGI-S and CGI-I are not dependent of each other, but instead scored separately for severity and improvement, these are only presented descriptively. Moreover, a review of the data indicated that both the self-report measures and the clinician-administered rating of overall health status were not always conducted in relation to signing the contract or the 12-month follow-up. Thus, it was decided that only data reported within one month before and after each occasion (pre and post) were to be used in order to prevent any other factors to affect the results. Further, mean differences, the summation and change of each variable (i.e., sum and sum change, including a presentation of percentages), and within-group effect sizes Cohen’s *d* are reported were applicable and relevant.

All analyses were performed on jamovi version 0.9.2.9 [21] and done a complete case basis, i.e., only those patients with available data are included (which means that the number of patients for each variable might vary). No attempts at dealing with missing data was in other words made, e.g., maximum likelihood estimation or multiple imputation. This was decided given the naturalistic design of the current study and availability of only two data points.

## Results

### Sample

Of the 182 patients that received PCA, only 56 (30.8%) provided their consent to participate in the research. In addition, only 42 of the original 182 patients (23.1%) had data that were possible to analyze with regard to hospitalization, 24 (13.2%) on EQ5D-3L and EQ5D VAS, and 20 (11.0%) on CGI.

### Hospitalization

Descriptive statistics for number of admissions to inpatient and involuntary care as well as inpatient and involuntary admission days can be observed in Table 2, including the sums and sum change for every variable. Results from the Wilcoxon Signed-Rank Test for each comparison are presented in Table 3. Overall, none of the variables demonstrated a significant difference between pre and post, with the exception of inpatient days, which exhibited a decrease by an average of 11.5 days, *p* < .01.

**Table 2:** Descriptive Statistics on Inpatient Days, Number of Admissions, Involuntary Admission Days, and Number of Involuntary Admissions

	***n***	***M***	***Mdn***	***IQR***	***SD***	Sum	Sum change (%)
Inpatient days (pre)	42	30.4	23.5	2.8	27.4	1278	
Inpatient days (post)	42	19.0	6.0	3.0	29.7	796	−37.7%
Number of admissions (pre)	42	3.0	2.0	48.3	3.2	126	
Number of admissions (post)	42	2.8	1.0	20.0	4.5	119	−5.6%
Involuntary admission days (pre)	42	19.5	0.0	21.5	32.7	819	
Involuntary admission days (post)	42	15.7	0.0	0.8	36.4	658	−19.7%
Number of involuntary admissions (pre)	42	1.0	0.0	1.0	1.7	42	
Number of involuntary admissions (post)	42	0.7	0.0	0.8	1.6	31	−26.2%

*n* Sample size, *M* Mean, *Mdn* Median, *IQR* Interquartile Range, *SD* Standard Deviation

**Table 3:** Results from the Wilcoxon Signed-Rank Test for Inpatient Days, Number of Admissions, Involuntary Admission Days, and Number of Involuntary Admissions

Paired variables	Statistic	***p***
Inpatient days	633	.003
Number of admissions	397	.319
Involuntary admission days	136	.498
Number of involuntary admissions	117	.168

### Self-report measures and clinician-administered rating of overall health status

Descriptive statistics for the EQ5D-3L, EQ5D VAS, CGI-S, and CGI-I are found in Table 4. Results from the Wilcoxon Signed-Rank Test for the EQ5D-3L and EQ5D VAS can be obtained in Table 5. Neither the EQ5D-3L and EQ5D VAS revealed a significant change from pre to post in terms of the patients’ self-rated health status. Moreover, with regard to the CGI-S and CGI-I, healthcare providers classified their patients to be, on average, 4.2 at pre (moderately ill), while the degree of improvement was rated as 4.1 at post (no change).

**Table 4:** Descriptive Statistics for the EQ5D-3L, EQ5D VAS, CGI-I, and CGI-S

	***n***	***M***	***Mdn***	***IQR***	***SD***
EQ5D-3L (pre)	24	0.7	0.8	0.5	0.3
EQ5D-3L (post)	24	0.8	0.8	0.2	0.2
EQ5D VAS (pre)	24	60.8	67.5	20.0	18.6
EQ5D VAS (post)	24	61.4	66.5	26.2	19.0
CGI-S	20	4.2	4.0	0.3	1.0
CGI-I	20	4.1	4.0	0.3	1.2

*VAS* Visual Analogue Scale, *CGI* Clinical Global Impression Scale, *n* Sample size, *M* Mean, *Mdn* Median, *IQR* Interquartile Range, *SD* Standard Deviation

**Table 5:** Results from the Wilcoxon Signed-Rank Tests for the EQ5D-3L and EQ5D VAS

Paired variables	Statistic	***p***	Cohen’s ***d***
EQ5D-3L	68.5	.106	0.4
EQ5D-3L VAS	110	.862	0.0

*VAS* Visual Analogue Scale

## Discussion

The current study explored the effects of introducing PCA to patients with schizophrenia spectrum diagnoses within Region Stockholm’s public healthcare and using a naturalistic within-group follow-up design of 12 months. The results demonstrated pre-post changes with regard to inpatient days, number of admissions, involuntary admission days, and number of involuntary admissions, i.e., a decrease between 5.6–37.7%. However, this was only statistically significant for inpatient days, exhibiting a mean decrease of 11.5 days, *p* < .01. Hence, although some trends can be observed, the benefits of allowing admissions to patients with schizophrenia spectrum diagnoses are inconclusive, at least in terms of hospitalization. These findings are somewhat in line with what has been found in prior research. In a similar study in Norway that followed 53 patients randomized to PCA or TAU for 12 months [17], inpatient days decreased from pre to post by a mean of 63.3 days. The reason for this difference is unclear, but could be related to what inclusion criteria were used or certain patient characteristics. It is possible that patients in the current study were less severely ill or had lower care needs for other reasons. It is also plausible that the typical patient receiving PCA changed over time as healthcare providers became more accustomed to its use. However, the lack of information in terms of the patients’ features in the two studies prevents further investigation. Meanwhile, a Danish matched-controlled study with 2532 patients comparing PCA (*n* = 422) to TAU (*n* = 2110) [10], self-referrals resulted in a mean increase of 0.92 admissions, but the study did not present any data on inpatient days. Neither of the studies demonstrated any significant difference between the conditions, making it difficult to infer that it is PCA per se that drives these effects. This is similar to a recent study employing a four-year pre-post naturalistic design in Norway, in which the total number of admissions increased and inpatient days decreased, but where regression to the mean effects are impossible to rule out [18]. Nonetheless, from a patient perspective these potential changes in hospitalization might be considered less relevant if it has a positive impact on empowerment, well-being, and symptom severity. Similarly, a decrease in inpatient days may not be desirable in itself as the purpose of PCA is to allow patients to admit themselves when preferred rather than when necessary. Future research should perhaps focus less on hospitalization and more on such benefits as the ability to manage everyday life.

As for self-report measures of well-being, no or small changes were observed in the current study, *d* = 0.0 and 0.4 on the EQ5D-3L and the EQ5D-VAS, neither exhibiting a significant change. In addition, using a clinician-administered rating of overall health status, CGI, healthcare providers evaluated the patients to be moderately ill at pre, while the degree of improvement was considered null at post, implying that no change occurred on these types of assessments. These results are hard to compare to previous research given that no study has used similar outcomes. Only one study of patients with schizophrenia spectrum diagnoses has previously used any self-report measure [22], but did not demonstrate any changes on the Patient Activation Measure, i.e., knowledge, skill, and confidence in self-management [23], the Recovery Assessment Scale, i.e., recovery from severe psychiatric conditions [24], or the Behavior and Symptom Identification Scale, i.e., symptom and functioning difficulties [25]. Furthermore, there were no differences between PCA and TAU on any of these variables. However, looking at other psychiatric disorders and using the World Health Organization Disability Assessment Schedule II (WHODAS-II) [26], the domains cognition, mobility, domestic responsibilities, and participation did demonstrate moderate positive changes for patients with personality disorders who self-harm and are suicidal [12], *d* = 0.42-0.72. Similarly, a significant increase of *d* = 0.78 was observed on the EQ5D-VAS and *d* = 0.45 on the Global Assessment of Functioning [27] was found for patients with anorexia nervosa [13]. Hence, the impact of PCA on other aspects than hospitalization is mixed and inconclusive, warranting additional research to get a better idea of how self-referrals affect patients.

There are a number of strengths and limitations that need to be discussed in relation to the results of the current study. Given its naturalistic design the external validity is considered high. No changes in normal procedures at the outpatient clinics or inpatient wards were made, except for introducing PCA, which suggest that the findings should be fairly generalizable to similar contexts, that is, comparable to how healthcare is organized in the Scandinavian countries. Also, the application of PCA was adapted from Norway [9, 17], which is the care model most widely used, that is, signing a contract with patients having the greatest care needs and allowing up to five inpatient days when requested [14]. This should make the results relatively analogous to previous research, although one notable exception is that a quarantine period of two weeks was not used in the current study [9, 10, 17], nor were other restrictions, e.g., limiting the total number of admissions per month [12].

However, the implications of the current study are restricted by a number of significant limitations. In addition to addressing their implications for the results that have been presented, recommendations on how to advance the research on PCA are provided. First, it lacked adequate statistical power to evaluate its effects. Few of the patients that were deemed eligible to receive PCA met all of the inclusion criteria for the current study and provided their consent to take part in the research. Also, data available at pre and post differed between variables, possibly due to inconsistencies surrounding data collection procedures. The sample size is nonetheless similar to that of the study from Norway (*n* = 53) and can be seen as a replication that can be included in a systematic review and meta-analysis [9, 17]. Yet, to fully understand the possible benefits of PCA for patients with schizophrenia spectrum diagnoses as well as other patient groups, future studies need to be much larger and the recruitment procedures will have to be more inclusive. Following the introduction of PCA in the current study, the Commissioner of Healthcare in Region Stockholm, who manages the publicly funded healthcare, has decided to provide all patients with severe psychiatric conditions access to PCA within the next years. This will eventually entail 47 inpatient wards and a variety of different patient groups, allowing a greater sample size and more sophisticated statistical analyses. Second, a number of characteristics in relation to the patients in the current study were not possible to examine, which limits the interpretation of the results. Without having a better picture of such aspects as cognitive impairment and motivational level it is unclear whether PCA is only utilized by a select group of patients. That is, it might be the case that those with greater cognitive resources are more apt to admit themselves when needed. Coming studies should try to explore this issue further, such as by looking into neuropsychological assessments, additional demographics, and support from significant others. Third, although the current study provided some additional information on the possible advantages of PCA by looking into self-rated well-being, it is still uncertain to what extent this care model could help patients [14]. Qualitative studies point to an increase in confidence and the ability to cope with life and to experience the option to self-refer as a safety net even though it is not used [28, 29]. Thus, it might exist other variables have been missed and need to be examined quantitatively, for example autonomy and quality of life, which should be included in future studies on PCA. Fourth, the current study did not use a random allocation in order to evaluate the effects against a comparator. This was however not feasible given the restrictions imposed by Region Stockholm’s public healthcare due to ethical concerns, but should be seen as imperative in future research of PCA to accurately determine its results. To date, none of the prior studies utilizing such a design has been able to distinguish a difference between PCA and TAU with regard to hospitalization [9, 12, 13, 17]. However, this should be investigated further by using other comparators. Seeing as signing a contract might constitute an intervention in itself by being perceived as a safety net [28, 29], similar procedures but without actually allowing patients to admit themselves to inpatient care could be a feasible control condition. This could be comprised of collaboratively setting up and signing a treatment plan, as well as having follow-up assessments at a similar frequency as PCA. This should help explore what drives the effects on hospitalization and be an ethical alternative to TAU or a waiting list. Fifth, the patients in the current study were followed up to one year, but determining the long-term effects of PCA is essential to determine its potential benefits. A recent study with a four-year follow-up suggests that results are maintained over time [18], but could however not rule out regression to the mean as a possible explanation for the results, implying that more research needs to be conducted on a long-term basis. Additional outcomes could also be included in this analysis, e.g., sick leave, other healthcare expenditures, and occupational status, in order to better understand how PCA might affect other relevant outcomes in everyday life. Lastly, the current study did not assess adherence or fidelity with regard the procedures surrounding the healthcare providers implementation of PCA. Hence, it is possible that local variations emerged with regard to how it was utilized and to whom it was offered. This might explain why the CGI-I indicated a moderate severity level for the patients that were analyzed, as well as the fact that data on hospitalization was positively skewed, i.e., a small number of patients had high care needs, which goes against the initial idea of offering PCA only to those with the greatest care needs. The Centre for Psychiatry Research at the Department of Clinical Neuroscience at Karolinska Institutet did introduce the care model to the outpatient and inpatient wards and provided additional assistance, but did not systematically follow-up on the actual implementation and adherence to inclusion criteria. Future studies should thus explore this more thoroughly, especially in relation to implementation science, i.e., studying the methods for promoting uptake and consolidation of research into healthcare practice and policy [30]. In addition, another interesting aspect would be to involve patients themselves to a greater degree in order to identify relevant outcomes.

In sum, the current study demonstrated an overall decrease in relation to inpatient days, involuntary admission days, and number of involuntary admissions that might indicate a trend towards a reduction in hospitalization, although only the change in inpatient days was statistically significant, suggesting that the results should be interpreted cautiously. Meanwhile, self-report measures on well-being and a clinician-administered rating of overall health status did not reveal any major changes. However, more research is warranted using a larger sample size, randomization, and additional outcomes to accurately explore the potential advantages of providing patients with the opportunity to admit themselves when needed. Furthermore, as the current study and most research on PCA has been conducted in the Scandinavian countries, generalizability of the results could be restricted to those contexts that have a similar healthcare system. Future research should therefore investigate its benefits in other settings.

## Data Availability

The datasets used and/or analyzed during the current study are available from the corresponding author upon reasonable request and authorization from the Region Stockholm’s public healthcare.

## Abbreviations

PCA: Patient-Controlled Admissions
TAU: Treatment as Usual
CGI: Clinical Global Impression Scale (S = Symptoms; I = Improvement)
VAS: Visual Analogue Scale
N: Sample size
M: Mean
Mdn: Median
IQR: Interquartile Range
SD: Standard Deviation
